# Anthropometric indicators of obesity in the prediction of high body fat in adolescents

**DOI:** 10.1016/j.rpped.2014.06.007

**Published:** 2015-03

**Authors:** Andreia Pelegrini, Diego Augusto Santos Silva, João Marcos Ferreira de Lima Silva, Leoberto Grigollo, Edio Luiz Petroski

**Affiliations:** a Universidade do Estado de Santa Catarina, Florianópolis, SC, Brazil; b Universidade Federal de Santa Catarina, Florianópolis, SC, Brazil; c Faculdade Leão Sampaio, Juazeiro do Norte, CE, Brazil; d Universidade do Oeste de Santa Catarina, Joaçaba, SC, Brazil

**Keywords:** Anthropometry, Body fat distribution, Students

## Abstract

**OBJECTIVE:**

To determine the anthropometric indicators of obesity in the prediction of high body fat in adolescents from a Brazilian State.

**METHODS:**

The study included 1,197 adolescents (15-17 years old). The following anthropometric measurements were collected: body mass (weight and height), waist circumference and skinfolds (triceps and medial calf). The anthropometric indicators analyzed were: body mass index (BMI), waist circumference (WC), waist-to-height ratio (WHtR) and conicity index (C-Index). Body fat percentage, estimated by the Slaughter et al equation, was used as the reference method. Descriptive statistics, U Mann-Whitney test, and ROC curve were used for data analysis.

**RESULTS:**

Of the four anthropometric indicators studied, BMI, WHtR and WC had the largest areas under the ROC curve in relation to relative high body fat in both genders. The cutoffs for boys and girls, respectively, associated with high body fat were BMI 22.7 and 20.1kg/m², WHtR 0.43 and 0.41, WC 75.7 and 67.7cm and C-Index 1.12 and 1.06.

**CONCLUSIONS:**

Anthropometric indicators can be used in screening for identification of body fat in adolescents, because they are simple, have low cost and are non-invasive.

## Introduction

Overweight has been considered an important public health problem worldwide.^1^ Evidence consistently reports that there is a greater likelihood of overweight and obese adolescents to become obese adults.^2^ In this context, obesity in childhood and in adolescence is considered an independent risk factor in the development of cardiovascular diseases in adulthood.^3^

Numerous methods have been used to assess body composition.^4^ Among indirect methods, hydrostatic weighing and dual energy X-ray absorptiometry (DEXA) stand out; however, they are more difficult to be used in large samples due to the high cost and the need for a qualified technical team for assessing the measurements.^5^ Among double indirect methods, anthropometry is considered a simple, rapid, inexpensive method that can be applied to a great number of individuals.^6^

Many anthropometric indicators have been proposed to diagnose the health risks taking into account the increased body fat.^7^ The most widely used is still the body mass index (BMI), but it has some limitations.^8^ However, other indicators have been recommended. Waist circumference (WC) is one of the measures proposed to achieve results closer to reality, since abdominal fat deposits also cause, alone, various health problems.^9^ The waist-to-height ratio (WHtR)^10^ and the conicity index (C-Index) have also been used as indicators to diagnose body fat. 

Some studies have been conducted with children and adolescents in order to analyze the performance of anthropometric indicators (BMI, WC, WHtR) in the diagnosis of excess body fat.^11^^-^ 14 In Brazil, few studies have investigated the ability of each indicator to detect excess body fat in adolescents,^15^^,^ 16 however, studies using anthropometric indicators to predict high blood pressure^17^ and hypertension stand out.^18^ Both in Brazil and in other countries, no studies investigating the C-Index for the prediction of high body fat were found. In this sense, there are discussions about what would be the best anthropometric index for predicting high body fat, regardless of sex, age and total body fat. Therefore, more empirical evidence is needed, especially in adolescence. Thus, this study aims to verify the diagnostic performance of anthropometric indicators of obesity in the prediction of high body fat in adolescents.

## Methods

This cross-sectional epidemiological study included schoolchildren aged 15-17 years enrolled in public schools (state and federal) in the Brazilian state of Santa Catarina. The study was approved by the Ethics Committee on Human Research of the Federal University of Santa Catarina (protocol number 372/2006) and University of Western Santa Catarina (protocol number 079/08). 

To conduct the survey, two regions were considered: 1) a survey was conducted in 2007 in Florianópolis, capital of the state of Santa Catarina, located in southern Brazil. Florianópolis has a population of approximately 420,000 inhabitants,^19^ and is considered one of the Brazilian cities with the highest human development index (HDI=0.875).^20^ The other region considered was the western region of Santa Catarina, one of the mesoregions of the state.^19^ The western region of Santa Catarina has an HDI of 0.807.^20^ Among the top 20 cities in quality of life in Brazil, five are from the western region of Santa Catarina, which has an estimated population of 25,322 inhabitants.^19^

The sample was calculated separately for each region. The following parameters were used: prevalence for the outcome of 50% (unknown prevalence), tolerable error of five percentage points, confidence level of 95%, and a delimitation effect of 1.5, adding 10% for possible losses/refusals. Thus, 634 adolescents in each region were evaluated, composing a total sample of 1,268 adolescents. 

In Florianópolis, the sampling process was determined in two stages: stratified by geographic region and conglomerate groups. In the first stage, the city was divided into five geographical regions: center, continent, east, north and south. The school with the largest number of students from each region was selected, and in each school, classes were randomly selected to represent a sample representative of the geographic area. In the second stage, all adolescents who were present in classroom on the day of data collection were invited to participate in the study.

In the Midwestern region of Santa Catarina, the sampling process was determined in two stages: stratified by public high schools and classes conglomerates. In the first stage, only schools with over 150 students were considered. Moreover, in cities with more than one teaching unit, we chose the one with the highest number of students. In the second stage, all adolescents who were present in classroom on the day of data collection were invited to participate in the study. 

For this investigation, we defined as eligible the students enrolled in public state schools, those present in the classroom on the day of data collection and those aged 15-17 years. The exclusion criteria were: (a) students either <15 or >17 years old; (b) students who did not bring the Free and Informed Consent Form (FICF) signed by parents and/or guardian; (c) students who refused to participate; (d) students who did not perform anthropometric measurements. 

Fieldwork was conducted by Physical Education teachers and students, trained to carry out all the necessary procedures in order to standardize data collection. School students were instructed on evaluations at least five days in advance. At that time, the FICF was presented and they were informed about the procedures for the tests. The data collection team was trained in order to standardize the anthropometric measurements. The technical error of measurement was not calculated, but the researcher responsible for the survey had extensive experience in anthropometric measurements and routinely performed the quality control of the team of evaluators.

Anthropometric body mass data - weight and height, waist circumference, triceps skinfold thickness (TSFT) and medial calf skinfold thickness (MCST) were measured according to standardized procedures.^21^ Body mass index (BMI) was calculated and ranked according to cutoff points for adolescents, which vary according to age and gender.^22^ Abdominal obesity was verified by measuring waist circumference. WHtR was assessed by the waist x height ratio in cm. C-Index was determined by measuring body mass (weight and height) and waist circumference, using the Valdez mathematical equation.^23^

Body fat was verified by the relative body fat - % BF,^24^ for boys and girls, using the sum (Σ2DC) of TSFT and MCST, as shown below:


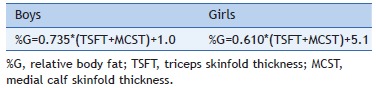


The cutoff points used for the classification of body fat were those recommended by Lohman,^25^ according to gender and age, in which values higher than 20 for boys and 25 for girls were considered high. 

Mean and standard deviation were used in the descriptive analysis of variables. The Kolmogorov-Smirnov test was used to verify data normality. Differences in the averages of variables between genders were analyzed by the Mann Whitney test. Association between anthropometric indicators and gender was assessed by the chi-square test. To evaluate the diagnostic performance of BMI, WHtR and C-Index in detecting excess body fat, the ROC curve analysis was applied. The diagnostic accuracy refers to the ability of BMI, WHtR and C-Index to discriminate adolescents with excess body fat from those without excess body fat. Areas under the ROC curve and confidence intervals were determined. To better determine the optimal critical values of anthropometric indicators with greater accuracy in the overweight detection, sensitivity and specificity were considered for each gender. The significance level was set at *p*<0.05. Analyses were performed using SPSS (Statistical Package for Social Sciences) 20.0 version and MedCalc.

## Results

The study showed a response rate of 94.4% (n=1,197), with 478 male and 719 female adolescents aged 15-17 years. The sample characteristics are presented in Table 1. Boys had higher body mass, height, WC, WHtR and C-Index, while girls had higher averages of TSFT, MCST, sum of two skinfolds (Σ2DC) and fat percentage (BF%) (*p*<0.05). 

**Table 1:**
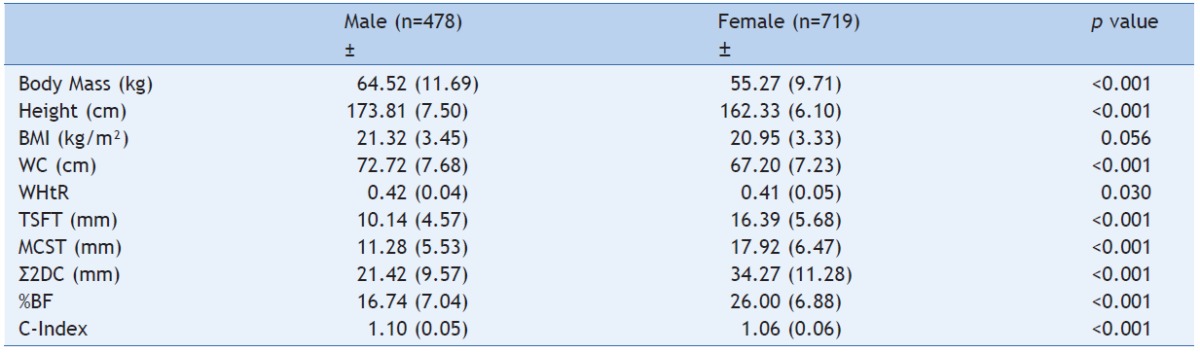
General characteristics of the sample ±.

BMI, body mass index; WC, waist circumference; WHtR, waist/height ratio; TSFT, triceps skinfold thickness; MCST, medial calf skinfold thickness; ∑2DC, sum of two skinfolds; % BF, relative body fat; C-Index, conicity index

The values of the area under the ROC curve, cutoff points, sensitivity and specificity are presented (Table 2) for all anthropometric indicators as discriminators of high relative body fat. All anthropometric indicators analyzed showed predictive ability to identify subjects with high body fat (i.e. lower limit of CI95% of the area under the ROC curve >0.50). BMI, WHtR and WC had greater ability to discriminate body fat in both genders compared to the C-Index (Table 2). 

**Table 2:**
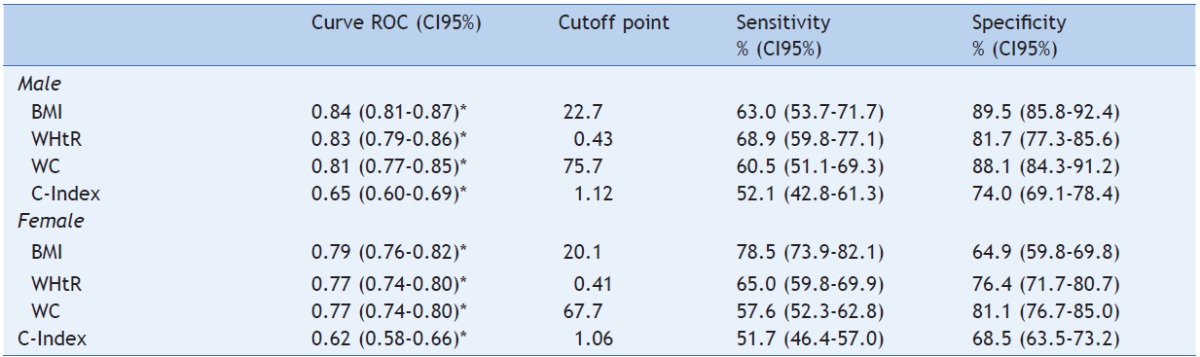
Diagnostic properties of anthropometric indicators of obesity to detect high body fat percentage in adolescents according to gender.

CI95%, confidence interval; BMI, body mass index; WC, waist circumference; WHtR, waist/height ratio; C-Index, conicity index. *: area under the ROC curve demonstrating discriminatory power for body fat (lower limit of CI95%>0.50).

**Figure 1:**
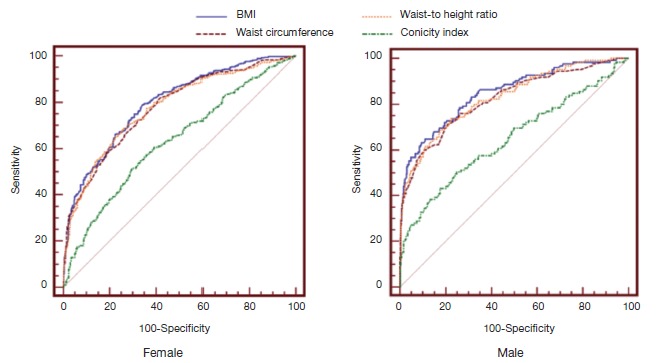
Area under the ROC curve of anthropometric indicators in predicting body fat in adolescents.

The areas under the ROC curve of anthropometric indicators in the prediction of body fat in adolescents can be observed in Figure 1. Significant differences were observed between the ROC curves in both genders, which show that the ROC curve for the C-Index has the lowest percentage under the curve when compared to BMI, WC and WHtR (*p*<0,05). 

## Discussion

All anthropometric indicators were able to diagnose excess body fat, as they showed the lowest limit of 95% of the area under the ROC curve up to 0.50. However, BMI, WHtR and WC had greater ability to discriminate body fat in both genders compared to the C-Index. These results show that not only indicators of general obesity (BMI), but also indicators of central obesity (WC, WHtR) can be used in adolescents to diagnose high body fat.

These results were similar to those presented by Brazilian adults in relation to the C-Index, which is an anthropometric indicator with low discriminatory power for health problems compared to other anthropometric indicators.^26^ The C-index was a good predictor for chronic non-communicable diseases.^27^

WC and WHtR had enough similarity to discriminate body fat in this study. A study conducted in southern Brazil also revealed that these anthropometric indicators showed similarity to predict blood hypertension.^26^ The similarity between these indicators lies in the fact that both deal with fat located in the central region.^10^ This study also reported that BMI was similar to WC and WHtR to detect the adiposity anthropometric indicator, which shows that during adolescence this measure may be useful for diagnosing obesity.^10^

The findings of this study have vital implications for the assessment of obesity among adolescents, since it reinforces the use of anthropometric indicators of obesity, which are relatively simple to be evaluated, as a discriminator of body fat. There is no doubt that the assessment of body composition by skinfold is more accurate than using anthropometric indicators, as shown by Nooyens et al*.* 28 However, the measurement of skinfolds requires trained evaluators to provide accurate measurements. Thus, the World Health Organization^29^ recommends the use of simpler anthropometric indicators of obesity to evaluate possible health risks. 

Research conducted with Spanish children and adolescents revealed that BMI, triceps skinfold thickness and WC were good anthropometric indicators in the diagnosis of total body fat assessed by the doubly labeled water method.^13^ In North American children and adolescents (5-18 years old), it was shown that both BMI and fat percentage (derived from skinfolds) are low cost, viable indicators that can be used for screening excess body adiposity.^30^ BMI and WC provided better diagnostic in screening obesity (measured by plethysmography) in adolescents than the waist-hip circumference ratio (WHtR) in Swedish adolescents.^14^ Based on the results of this study and those found in literature, it could be inferred that for adolescents anthropometric indicators of general obesity and central obesity are both good predictors of high body fat.

The best cutoff point for BMI to detect the emergence of high body fat was 22.7kg/m² for boys and 20.1kg/m² for girls. Usually, the cutoff points for BMI in adolescents vary according to gender and age.^22^^,^ 31 A study that developed cutoff points for BMI in a sample of Brazilian adolescents reported that in the age group of this study (14-17 years old), BMI for overweight ranged from 21.7kg/m² to 23.6kg/m² for males and from 22.8kg/m² to 24.8kg/m² for females. For obesity, the mentioned study reported that for males the cutoff point for BMI ranged from 27.5kg/m² to 28.7kg/m² and for females the cuttoff point ranged from 27.5kg/m² to 29.6kg/m². In the study by Cole et al,^22^ who developed cutoff points for BMI in a sample of children and adolescents from six countries (Brazil, Great Britain, Hong Kong, the Netherlands, Singapore and the United States), BMI for overweight ranged from 22.6kg/m² to 24.5kg/m² for males and 23.3kg/m² to 24.7kg/m² for females. For obesity, the cutoff point for BMI ranged from 27.6kg/m² to 29.4kg/m² for males and the cuttoff point for females ranged from 28.6kg/m² to 29.7kg/m². It was observed that the cutoff point for BMI for males in this study is in the overweight range of other studies.^22,31^ Moreover, the cutoff point for BMI in this study for females is below those found in literature to detect overweight. One possible explanation for these discrepancies may be related to ethnic and cultural differences in Brazilian adolescents that may influence BMI. 

As for WC, it was observed that the best cutoff point to detect the emergence of high body fat was 75.7cm and 67.7cm for boys and girls, respectively. Fernandez et al,^32^ when developing cutoff points for WC in a sample representative of children and adolescents of different ethnicities (African Americans, European Americans and Mexican Americans) found that, in the age group of this study, WC ranged from 79.4cm to 87.0cm for males and from 78.3cm to 85.5cm for females. It was also observed that the cutoff points found for adolescents of this investigation are lower than those of other studies.^32^ Evidence shows that, among the anthropometric indicators, WC had the best performance in the diagnosis of obesity in children and adolescents.^11^^,^ 14

Regarding WHtR, the best cutoff point to detect the emergence of high body fat was 0.43cm and 0.41cm for males and females, respectively. Studies conducted with Italian^33^ and African adolescents^34^ found that the best diagnostic value of WHtR for metabolic risk was 0.41, which is similar to the findings of this study, and lower than what is internationally proposed (0.50). Moreover, this indicator has been considered one of the best in the evaluation of central fat distribution, and it is associated with various cardiovascular risk factors.^10^ As for predicting high body fat, it is possible to observe that WHtR has been considered a simple, easy-to-use, accurate indicator, with high applicability in screening overweight and obesity in children and adolescents.^12^

The best cutoff point for the C-Index was 1.12 for boys and 1.06 for girls. Publications on the prediction of high body fat through the C-Index were not found, which makes it difficult to compare the results found in this study. However, cutoff points for the C-Index were developed to detect high blood pressure (boys=1.13 and girls=1.14), high levels of total cholesterol (boys=1.10) and low levels of HDL-c (girls=1.10).^35^

Among the limitations of the study, the use of double indirect measures (skinfold) to establish the criterion measure of body fat can be highlighted; however, in the assessment of nutritional status and body composition in children and adolescents, such measures are commonly used and recommended by health agencies.^29^

According to the findings of this study, it could be concluded that anthropometric indicators can be used in screening to identify high body fat in adolescents for being a simple, inexpensive and non-invasive method. These findings reinforce the possibility of using anthropometric indicators as an alternative to evaluate adolescents, through simple, replicable and reliable criteria, with high sensitivity and specificity at low cost, which allows greater range in the scope of monitoring nutritional and health status among adolescents.
