# Supplementary material for: Anthropometric indicators of obesity in the prediction of high body fat in adolescents
Source: Rev Paul Pediatr. 2015 Mar;33(1):56–62. doi: 10.1016/j.rpped.2014.06.007 (PMC4436957; doi:10.1016/j.rpped.2014.06.007)
Supplement: Translation

# Translation: Indicadores antropométricos de obesidade na predição de gordura corporal elevada em adolescentes

**Authors:** Andreia Pelegrini, Diego Augusto Santos Silva, João Marcos Ferreira de Lima Silva, Leoberto Grigollo, Edio Luiz Petroski

**Affiliations:** a Universidade do Estado de Santa Catarina (Udesc), Florianópolis, SC, Brasil; b Universidade Federal de Santa Catarina (UFSC), Florianópolis, SC, Brasil; c Faculdade Leão Sampaio (Fals), Juazeiro do Norte, CE, Brasil; d Universidade do Oeste de Santa Catarina (Unoesc), Joaçaba, SC, Brasil

**Keywords:** Antropometria, Distribuição de gordura corporal, Estudantes

## Abstract

**OBJETIVO:**

Determinar os indicadores antropométricos de obesidade na predição da gordura corporal elevada em adolescentes de um estado brasileiro.

**MÉTODOS:**

O estudo incluiu 1.197 adolescentes (15-17 anos). As seguintes medidas antropométricas foram coletadas: massa corporal e estatura, perímetro da cintura e dobras cutâneas (tríceps e perna medial). Os indicadores antropométricos analisados foram: índice de massa corporal (IMC), perímetro da cintura (PC), razão cintura-estatura (RCE) e índice de conicidade (IC). A gordura corporal elevada, estimada pela equação de Slaughter et al., foi usada como método de referência. Estatística descritiva, teste U de Mann-Whitney e curva ROC foram usadas para a análise dos dados.

**RESULTADOS:**

Dos quatro indicadores antropométricos estudados, o IMC, a RCE e o PC tiveram as maiores áreas sob a curva ROC em relação à gordura corporal elevada relativa em ambos os sexos. Os pontos de corte para os rapazes e as moças, respectivamente, associados com gordura corporal elevada foram IMC 22,7 e 20,1 kg/m^2^, RCE 0,43 e 0,41, PC 75,7 e 67,7 cm e IC 1,12 e 1,06.

**CONCLUSÕES:**

Os indicadores antropométricos podem ser usados como ferramenta para identificação da gordura corporal em adolescentes, por serem um método simples, de baixo custo e não invasivo.

## Introdução

O excesso de peso tem sido considerado um importante problema de saúde pública em todo o mundo.^1^ Evidências mostram de forma consistente que há uma maior probabilidade de adolescentes com sobrepeso e obesos se tornarem adultos obesos.^2^ Nesse contexto, a obesidade na infância e adolescência é considerada um fator de risco independente no desenvolvimento de doenças cardiovasculares na idade adulta.^3^

Diversos métodos têm sido usados para avaliar a composição corporal.^4^ Entre os métodos indiretos, a pesagem hidrostática e a absorciometria com raios-X de dupla energia (Dexa) se destacam; no entanto, elas são mais difíceis de ser usadas em um grande número de amostras devido ao elevado custo e à necessidade de uma equipe técnica qualificada para avaliar as medidas.^5^ Entre os métodos indiretos duplos, a antropometria é considerada um método simples, rápido e de baixo custo, que pode ser aplicado em um grande número de indivíduos.^6^

Muitos indicadores antropométricos têm sido propostos para diagnosticar os riscos para a saúde levando em consideração o aumento da gordura corporal.^7^ O mais usado ainda é o índice de massa corporal (IMC), que, contudo, tem algumas limitações.^8^ No entanto, outros indicadores têm sido recomendados. O perímetro da cintura (PC) é uma das medidas propostas para atingir resultados mais próximos do real, já que os depósitos de gordura abdominal também causam, sozinhos, vários problemas de saúde.^9^ A razão cintura-estatura (RCE)^10^ e o índice de conicidade (IC) também têm sido usados como indicadores para diagnosticar a gordura corporal.

Alguns estudos têm sido feitos em crianças e adolescentes a fim de analisar o desempenho de indicadores antropométricos (IMC, PC, RCE) no diagnóstico do excesso de gordura corporal.^11^^,^ 12 ^,^ 13 ^and^ 14 No Brasil, poucos estudos investigaram a capacidade de cada indicador de detectar o excesso de gordura corporal em adolescentes.^15^ ^and^ 16 No entanto, estudos que usaram indicadores antropométricos para prever pressão arterial elevada^17^ e hipertensão se destacam.^18^ Tanto no Brasil quanto em outros países não foram encontrados estudos que analisaram o IC para a predição de gordura corporal elevada. Nesse sentido, há discussões sobre qual seria o melhor índice antropométrico para predizer a gordura corporal elevada, independentemente do sexo, da idade e da gordura corporal total. Portanto, são necessárias mais evidências empíricas, especialmente na adolescência. Dessa forma, este estudo tem como objetivo verificar o desempenho diagnóstico dos indicadores antropométricos de obesidade na predição de gordura corporal elevada em adolescentes.

## Método

Este estudo epidemiológico transversal incluiu escolares de 15-17 anos matriculados em escolas públicas (estaduais e federais) no estado brasileiro de Santa Catarina. O estudo foi aprovado pelo Comitê de Ética em Pesquisa com Seres Humanos da Universidade Federal de Santa Catarina (número de protocolo 372/2006) e da Universidade do Oeste de Santa Catarina (número de protocolo 079/08).

Para fazer a pesquisa, duas regiões foram consideradas: 1) uma pesquisa foi feita em 2007, em Florianópolis, capital do Estado de Santa Catarina, localizado no sul do Brasil. Florianópolis tem uma população de 420.000 habitantes^19^ e é considerada uma das cidades brasileiras com o maior índice de desenvolvimento humano (IDH=0,875).^20^ A outra região considerada foi a Região Oeste de Santa Catarina, uma das mesorregiões do estado.^19^ A Região Oeste de Santa Catarina tem um IDH de 0,807.^20^ Entre as 20 melhores cidades em qualidade de vida no Brasil, cinco são da Região Oeste de Santa Catarina, que tem uma população estimada de 25.322 habitantes.^19^

A amostra foi calculada separadamente para cada região. Foram usados os seguintes parâmetros: prevalência para o desfecho de 50% (prevalência desconhecida), erro tolerável de cinco pontos percentuais, nível de confiança de 95% e um efeito de delimitação de 1,5, com a adição de 10% para possíveis perdas/recusas. Assim, foram avaliados 634 adolescentes em cada região, que compuseram uma amostra total de 1.268 adolescentes.

Em Florianópolis, o processo de amostragem foi feito em duas etapas: estratificado por regiões geográficas e grupos de conglomerados. Na primeira etapa, a cidade foi dividida em cinco regiões geográficas: Centro, Continente, Leste, Norte e Sul. A escola com o maior número de estudantes de cada região foi selecionada e, em cada escola, as classes foram escolhidas aleatoriamente para representar uma amostra significativa da área geográfica. Na segunda etapa, todos os adolescentes que estavam presentes em sala de aula no dia da coleta de dados foram convidados a participar do estudo.

Na Região Centro-Oeste de Santa Catarina, o processo de amostragem foi determinado em duas etapas: estratificado por colégios públicos e conglomerados de classes. Na primeira etapa, foram consideradas apenas as escolas com mais de 150 alunos. Além disso, em cidades com mais de uma unidade de ensino, escolhemos aquela com o maior número de alunos. Na segunda etapa, todos os adolescentes que estavam presentes em sala de aula no dia da coleta de dados foram convidados a participar do estudo.

Para essa pesquisa, foram definidos como elegíveis os alunos matriculados em escolas públicas estaduais, aqueles presentes na sala de aula no dia da coleta de dados e aqueles entre 15-17 anos. Os critérios de exclusão foram: (a) os alunos com <15 ou >17 anos; (b) os alunos que não trouxeram o Termo de Consentimento Livre e Esclarecido (TCLE) assinado pelos pais e/ou responsável; (c) os alunos que se recusaram a participar; (d) os alunos que não fizeram medidas antropométricas.

O trabalho de campo foi feito por professores e alunos de educação física, treinados para fazer todos os procedimentos necessários, com o objetivo de padronizar a coleta de dados. Os estudantes das escolas foram instruídos sobre as avaliações com pelo menos cinco dias de antecedência. Nesse momento, o TCLE foi apresentado e eles foram informados sobre os procedimentos para os testes. A equipe de coleta de dados foi treinada a fim de padronizar as medidas antropométricas. O erro técnico da medida não foi calculado, mas o pesquisador responsável pelo levantamento tinha vasta experiência em medidas antropométricas e fez o controle de qualidade da equipe de avaliadores rotineiramente.

Os dados antropométricos de massa corporal - peso e altura, perímetro da cintura, dobra cutânea tricipital (DCT) e dobra cutânea da panturrilha medial (DCPM) foram medidos de acordo com procedimentos padronizados.^21^ O índice de massa corporal (IMC) foi calculado e classificado de acordo com pontos de corte para adolescentes, que variam de acordo com idade e sexo.^22^ A obesidade abdominal foi verificada por meio da medição do perímetro da cintura. A RCE foi avaliada pela relação cintura x estatura em cm. O IC foi determinado pela medida de massa corporal (peso e altura) e circunferência da cintura, com o uso da equação matemática de Valdez.^23^

A gordura corporal foi verificada pela gordura corporal relativa -%GC,^24^ para meninos e meninas, com o uso da somatória (S2DC) DCT e DCPM, como mostrado abaixo:



Os pontos de corte usados para a classificação da gordura corporal foram os recomendados por Lohman,^25^ de acordo com sexo e idade, em que valores acima de 20 para meninos e 25 para meninas foram considerados altos.

A média e o desvio-padrão foram usados na análise descritiva das variáveis. O teste de Kolmogorov-Smirnov foi usado para verificar a normalidade dos dados. As diferenças nas médias das variáveis entre os sexos foram analisadas pelo teste de Mann-Whitney. A associação entre os indicadores antropométricos e sexo foi avaliada pelo teste do qui-quadrado. Para avaliar o desempenho diagnóstico do IMC, RCE e IC na detecção de excesso de gordura corporal, foi aplicada a análise da curva ROC. A precisão do diagnóstico refere-se à capacidade de IMC, RCE e IC de discriminar os adolescentes com excesso daqueles sem excesso de gordura corporal. Foram determinadas as áreas sob as curvas ROC, bem como os intervalos de confiança. Para melhor determinar os valores críticos ideais dos indicadores antropométricos com maior acurácia na detecção do excesso de peso, a sensibilidade e especificidade foram consideradas para cada sexo. O nível de significância foi estabelecido em *p*<0,05. As análises foram feitas com o programa SPSS (Statistical Package for Social Sciences) versão 20.0 e o MedCalc.

## Resultados

O estudo mostrou uma taxa de resposta de 94,4% (n=1197), com 478 adolescentes do sexo masculino e 719 do feminino entre 15-17 anos. As características da amostra são apresentadas na tabela 1. Os meninos apresentaram maior massa corporal, estatura, PC, RCE e IC, enquanto as meninas apresentaram médias mais elevadas de DCT, DCPM, somatório de dobras cutâneas (S2DC) e percentual de gordura corporal (%GC) (*p*<0,05).

**Tabela 1:** Características gerais da amostra em média ± desvio padrão

	Sexo masculino (n=478)	Sexo feminino (n=719)	Valor de *p*
	±	±	
Massa corporal (Kg)	64,52 (11,69)	55,27 (9,71)	<0,001
Estatura (cm)	173,81 (7,50)	162,33 (6,10)	<0,001
IMC (kg/m^2^)	21,32 (3,45)	20,95 (3,33)	0,056
PC (cm)	72,72 (7,68)	67,20 (7,23)	<0,001
RCE	0,42 (0,04)	0,41 (0,05)	0,030
DCP (mm)	10,14 (4,57)	16,39 (5,68)	<0,001
DCPM(mm)	11,28 (5,53)	17,92 (6,47)	<0,001
S2DC (mm)	21,42 (9,57)	34,27 (11,28)	<0,001
%GC	16,74 (7,04)	26,00 (6,88)	<0,001
IC	1,10 (0,05)	1,06 (0,06)	<0,001

IMC, índice de massa corporal; PC, perímetro da cintura; WHtR, razão cintura/estatura; DCT, dobra cutânea tricipital; DCPM, dobra cutânea panturilha medial; S2DC, somatório das dobras; % GC, gordura corporal relativa; IC, Índice de conicidade.

Os valores da área sob a curva ROC, os pontos de corte, a sensibilidade e especificidade são apresentados (tabela 2) para todos os indicadores antropométricos como discriminadores de gordura corporal relativa elevada. Todos os indicadores antropométricos analisados apresentaram capacidade preditiva para identificar indivíduos com gordura corporal elevada (isto é, o limite inferior do IC95% da área sob a curva ROC > 0,50). IMC, RCE e PC tiveram maior capacidade de discriminar a gordura corporal em ambos os sexos, em comparação com o IC (tabela 2).

**Tabela 2:** Propriedades diagnósticas dos indicadores antropométricos de obesidade para detectar percentual de gordura corporal elevada em adolescentes de acordo com o gênero

Indicadores antropométricos	Curva ROC (IC 95%)	Ponto de corte	Sensibilidade % (IC 95%)	Especificidade % (IC 95%)
*Sexo masculino*				
IMC	0,84 (0,81-0,87)*	22,7	63,0 (53,7-71,7)	89,5 (85,8-92,4)
RCE	0,83 (0,79-0,86)*	0,43	68,9 (59,8-77,1)	81,7 (77,3-85,6)
PC	0,81 (0,77-0,85)*	75,7	60,5 (51,1-69,3)	88,1 (84,3-91,2)
IC	0,65 (0,60-0,69)*	1,12	52,1 (42,8-61,3)	74,0 (69,1-78,4)

*Sexo feminino*				
IMC	0,79 (0,76-0,82)*	20,1	78,5 (73,9-82,1)	64,9 (59,8-69,8)
RCE	0,77 (0,74-0,80)*	0,41	65,0 (59,8-69,9)	76,4 (71,7-80,7)
PC	0,77 (0,74-0,80)*	67,7	57,6 (52,3-62,8)	81,1 (76,7-85,0)
IC	0,62 (0,58-0,66)*	1,06	51,7 (46,4-57,0)	68,5 (63,5-73,2)

IC 95%, intervalo de confiança; IMC, índice de massa corporal; PC, perímetro da cintura; RCE, relação cintura/estatura; IC, índice de conicidade

* : área sob a curva ROC que demonstra poder discriminatório para gordura corporal (limite inferior do IC 95% > 0,50).

As áreas sob a curva ROC dos indicadores antropométricos na predição de gordura corporal em adolescentes podem ser observadas na figura 1. Foram observadas diferenças significativas entre as curvas ROC em ambos os sexos, que mostram que a curva ROC para o IC tem o menor percentual abaixo da curva em relação ao IMC, ao PC e à RCE (*p*<0,05).

**Figura 1:** Área sob a curva ROC de indicadores antropométricos para predizer a gordura corporal em adolescentes.

## Discussão

Todos os indicadores antropométricos foram capazes de diagnosticar o excesso de gordura corporal, uma vez que mostraram o limite mais baixo de 95% da área sob a curva ROC até 0,50. Entretanto, IMC, RCE e PC tiveram maior capacidade de discriminar a gordura corporal em ambos os sexos, em comparação com o IC. Esses resultados mostram que não apenas os indicadores de obesidade generalizada (IMC), mas também indicadores de obesidade central (PC, RCE), podem ser usados para diagnosticar a gordura corporal elevada em adolescentes.

Esses resultados foram semelhantes aos apresentados por adultos brasileiros em relação ao IC, que é um indicador antropométrico com baixo poder discriminatório para problemas de saúde em comparação com outros indicadores antropométricos.^26^ O IC foi um bom preditor para doenças crônicas não transmissíveis.^27^

O PC e a RCE apresentaram similaridade suficiente para discriminar a gordura corporal neste estudo. Um estudo feito no sul do Brasil também revelou que esses indicadores antropométricos apresentavam similaridade para prever hipertensão arterial.^26^ A semelhança entre esses indicadores está no fato de que ambos avaliam a gordura localizada na região central.^10^ Este estudo também relatou que o IMC foi semelhante ao PC e à RCE em detectar o indicador antropométrico de adiposidade, o que mostra que durante a adolescência essa medida pode ser útil para o diagnóstico de obesidade.^10^

Os resultados deste estudo têm implicações importantes para a avaliação da obesidade entre os adolescentes, uma vez que reforçam o uso de indicadores antropométricos de obesidade, relativamente simples de serem avaliados, como um discriminador de gordura corporal. Não há dúvida de que a avaliação da composição corporal por dobras cutâneas é mais precisa do que o uso de indicadores antropométricos, como mostrado por Nooyens et al*.* 28 No entanto, a medida de dobras cutâneas exige avaliadores treinados para fornecer medições precisas. Assim, a Organização Mundial da Saúde 29 recomenda o uso de indicadores antropométricos de obesidade mais simples para avaliar possíveis riscos à saúde.

Pesquisa feita com crianças e adolescentes espanhóis revelou que o IMC, a prega cutânea tricipital e o PC foram bons indicadores antropométricos no diagnóstico da gordura corporal total avaliada pelo método da água duplamente marcada.^13^ Em crianças e adolescentes americanos (cinco-18 anos) foi demonstrado que tanto o IMC quanto o percentual de gordura (derivado das dobras cutâneas) são indicadores viáveis e de baixo custo e que podem ser usados para a triagem do excesso de gordura corporal.^30^ O IMC e o PC resultaram em melhor diagnóstico na triagem da obesidade (medida por pletismografia) em adolescentes do que a relação cintura-quadril (RCQ) em adolescentes suecos.^14^ Com base nos resultados deste estudo e naqueles encontrados na literatura, pode-se inferir que, para os adolescentes, os indicadores antropométricos de obesidade geral e obesidade central são ambos bons preditores de gordura corporal elevada.

O melhor ponto de corte de IMC para detectar o aparecimento de gordura corporal elevada foi de 22,7 kg/m^2^ para os meninos e 20,1 kg/m^2^ para as meninas. Normalmente, os pontos de corte para IMC em adolescentes variam de acordo com sexo e idade.^22^ ^and^ 31 Um estudo que desenvolveu pontos de corte para o IMC em uma amostra de adolescentes brasileiros relatou que na faixa do estudo (14-17 anos) o IMC de sobrepeso variou de 21,7 kg/m^2^ a 23,6 kg/m^2^ para homens e de 22,8 kg/m^2^ a 24,8 kg/m^2^ para mulheres. Para a obesidade, o estudo mencionado relatou que no sexo masculino o ponto de corte para o IMC variou de 27,5 kg/m^2^ a 28,7 kg/m^2^ e para as mulheres de 27,5 kg/m^2^ a 29,6 kg/m^2^. No estudo de Cole et al.,^22^ que desenvolveram pontos de corte para o IMC em uma amostra de crianças e adolescentes de seis países (Brasil, Grã-Bretanha, Hong Kong, Holanda, Singapura e Estados Unidos), o IMC de sobrepeso variou de 22,6 kg/m^2^ a 24,5 kg/m^2^ para o sexo masculino e 23,3 kg/m^2^ a 24,7 kg/m^2^ para o feminino. Para a obesidade, o ponto de corte para o IMC foi de 27,6 kg/m^2^ a 29,4 kg/m^2^ para os homens e o ponto de corte para as mulheres variou de 28,6 kg/m^2^ a 29,7 kg/m^2^. Observou-se que o ponto de corte do IMC para o sexo masculino neste estudo está na faixa de sobrepeso de outros estudos.^22^ ^and^ 31 Além disso, o ponto de corte para o IMC neste estudo para as mulheres é inferior àqueles encontrados na literatura para detectar excesso de peso. Uma possível explicação para essas discrepâncias pode estar relacionada às diferenças étnicas e culturais em adolescentes brasileiros que podem influenciar o IMC.

Quanto ao PC, observou-se que o melhor ponto de corte para detectar o aparecimento de gordura corporal elevada foi 75,7 cm e 67,7 cm para meninos e meninas, respectivamente. Fernandez et al.,^32^ ao desenvolver os pontos de corte para PC em uma amostra representativa de crianças e adolescentes de diferentes etnias (negros americanos, europeus americanos e mexicanos americanos) constatou que, na faixa etária do estudo, o PC variou de 79,4 cm a 87 cm para os homens e de 78,3 cm a 85,5 cm para as mulheres. Observou-se também que os pontos de corte encontrados para adolescentes desta pesquisa são menores do que os de outros estudos.^32^ Evidências mostram que, entre os indicadores antropométricos, o PC teve o melhor desempenho no diagnóstico da obesidade em crianças e adolescentes.^11^ ^and^ 14

Em relação à RCE, o melhor ponto de corte para detectar o aparecimento de gordura corporal elevada foi 0,43 cm e 0,41 cm para homens e mulheres, respectivamente. Estudos feitos com adolescentes italianos^33^ e africanos^34^ descobriram que o melhor valor diagnóstico de RCE para risco metabólico foi de 0,41, semelhante ao resultado deste estudo e inferior ao proposto internacionalmente (0,50). Além disso, esse indicador foi considerado um dos melhores na avaliação da distribuição da gordura central e está associado a vários fatores de risco cardiovascular.^10^ Quanto à previsão de gordura corporal elevada, é possível observar que a RCE foi considerada um indicador simples, fácil de usar e preciso, com alta aplicabilidade na triagem de sobrepeso e obesidade em crianças e adolescentes.^12^

O melhor ponto de corte para o IC foi de 1,12 para meninos e 1,06 para meninas. Não foram encontradas publicações sobre a predição de gordura corporal elevada por meio do IC, o que torna difícil a comparação com os resultados encontrados neste estudo. No entanto, os pontos de corte para o IC foram desenvolvidos para detectar a pressão arterial elevada (meninos = 1,13 e meninas = 1,14), níveis elevados de colesterol total (meninos = 1,10) e baixos níveis de HDL-c (meninas = 1,10).^35^

Entre as limitações do estudo, pode-se destacar o uso de medidas indiretas duplas (dobras cutâneas) para estabelecer o critério de medida de gordura corporal; no entanto, na avaliação do estado nutricional e da composição corporal em crianças e adolescentes, tais medidas são comumente usadas e recomendadas por agências de saúde.^29^

De acordo com os resultados deste estudo, pode-se concluir que os indicadores antropométricos podem ser usados na triagem para identificar a gordura corporal elevada em adolescentes por ser um método simples, barato e não invasivo. Esses achados reforçam a possibilidade do uso de indicadores antropométricos como uma opção para avaliar adolescentes, por meio de critérios simples, reprodutíveis e confiáveis, com alta sensibilidade e especificidade, a um baixo custo, o que permite maior alcance no âmbito da monitoração do estado nutricional e de saúde entre os adolescentes.
